# Understanding the mechanisms of ventilator-induced lung injury using animal models

**DOI:** 10.1186/s40635-023-00569-5

**Published:** 2023-11-27

**Authors:** Pedro Leme Silva, Martin Scharffenberg, Patricia Rieken Macedo Rocco

**Affiliations:** 1 Laboratory of Pulmonary Investigation, Carlos Chagas Filho Biophysics Institute, Federal University of Rio de Janeiro, Centro de Ciências da Saúde, Avenida Carlos Chagas Filho, 373, Bloco G-014, Ilha Do Fundão, Rio de Janeiro, RJ 21941-902 Brazil; 2 Department of Anesthesiology and Intensive Care Medicine, Pulmonary Engineering Group, University Hospital Carl Gustav Carus at Technische Universität Dresden, Dresden, Germany

**Keywords:** Biotrauma, Inflammation, Mechanical power, Atelectasis, Overdistension

## Abstract

Mechanical ventilation is a life-saving therapy in several clinical situations, promoting gas exchange and providing rest to the respiratory muscles. However, mechanical ventilation may cause hemodynamic instability and pulmonary structural damage, which is known as ventilator-induced lung injury (VILI). The four main injury mechanisms associated with VILI are as follows: barotrauma/volutrauma caused by overstretching the lung tissues; atelectrauma, caused by repeated opening and closing of the alveoli resulting in shear stress; and biotrauma, the resulting biological response to tissue damage, which leads to lung and multi-organ failure. This narrative review elucidates the mechanisms underlying the pathogenesis, progression, and resolution of VILI and discusses the strategies that can mitigate VILI. Different static variables (peak, plateau, and driving pressures, positive end-expiratory pressure, and tidal volume) and dynamic variables (respiratory rate, airflow amplitude, and inspiratory time fraction) can contribute to VILI. Moreover, the potential for lung injury depends on tissue vulnerability, mechanical power (energy applied per unit of time), and the duration of that exposure. According to the current evidence based on models of acute respiratory distress syndrome and VILI, the following strategies are proposed to provide lung protection: keep the lungs partially collapsed (SaO_2_ > 88%), avoid opening and closing of collapsed alveoli, and gently ventilate aerated regions while keeping collapsed and consolidated areas at rest. Additional mechanisms, such as subject-ventilator asynchrony, cumulative power, and intensity, as well as the damaging threshold (stress–strain level at which tidal damage is initiated), are under experimental investigation and may enhance the understanding of VILI.

## Take-home message

Although mechanical ventilation can improve gas exchange and reduce the work of breathing, it may cause ventilator-induced lung injury (VILI). This narrative review elucidates the mechanisms underlying the pathogenesis, progression, and resolution of VILI, and discusses strategies that can mitigate VILI. Different static variables (peak, plateau, driving pressures, positive end-expiratory pressure, and tidal volume) and dynamic variables (respiratory rate, airflow amplitude and profile, and inspiratory time fraction) can contribute to VILI. Additional concepts (mechanical power and subject-ventilator asynchrony) that are currently under investigation are discussed.

According to the current experimental evidence, the following strategies are proposed to provide lung protection: keep the lungs partially collapsed (SaO_2_ > 88%), avoid opening and closing collapsed alveoli, and gently ventilate aerated regions while keeping collapsed and consolidated areas at rest. In addition, new mechanisms such as cumulative power and intensity, as well as damaging threshold (stress-strain level at which tidal damage is initiated) are under experimental investigation and may enhance the understanding of VILI.

## Background

Although mechanical ventilation provides benefits in many clinical situations, it can cause pulmonary structural damage [1], known as ventilator-induced lung injury (VILI), and hemodynamic instability [2]. This is in line with a series of potential harmful effects of mechanical ventilation, including increases in inflammatory infiltration and vascular permeability, hyaline membrane formation, and pulmonary edema. Death may occur during mechanical ventilation even with satisfactory blood gas exchange [3, 4].

The four main injury mechanisms associated with VILI are as follows: barotrauma/volutrauma caused by overstretching the lung tissues; atelectrauma, caused by repeated opening and closing of the alveoli resulting in shear stress; and biotrauma, the resulting biological response to tissue damage, which leads to lung and multi-organ failure [5].

Different static variables (peak, plateau, and driving pressures, positive end-expiratory pressure, and tidal volume) and dynamic variables (respiratory rate [RR], airflow amplitude, and inspiratory time fraction) can contribute to these mechanisms of VILI. Moreover, the potential for lung injury depends on tissue vulnerability, the energy applied per unit of time (mechanical power), and the duration of that exposure [6, 7]. This narrative review discusses the advantages and limitations of experimental VILI, elucidates the mechanisms underlying the pathogenesis, progression, and resolution of VILI, and analyzes the strategies that can mitigate VILI.

### Advantages and limitations of experimental VILI

Experimental models allow researchers to investigate the mechanisms of VILI, which would be impossible and/or unethical in humans. Thus, different models of VILI have been developed and studied in diverse animal species in the last decades [8]. Some of the most common VILI models are summarized in Table 1. However, animal studies present some limitations that need to be considered in planning, conducting, and interpreting the results [9]. There are several physiologic and anatomic differences between humans and animals, which may influence the pulmonary response to an acute stimulus [10]. In this context, the RR is higher in mice (250–300 breaths per minute [bpm]) and rats (80–120 bpm) compared with humans (12–16 bpm). In addition, the lung structure of mice does not include bronchial arteries, and the size of the alveolus and the thickness of the alveolar-capillary membrane are smaller than those observed in rats and humans. Unlike the human lung, mice and rats have a monopodial airway branching pattern, whereas the human bronchial tree shows divisions with a dichotomic pattern (each bronchus is divided into two distal bronchi). In terms of inflammatory response, which is important during the development of VILI in animals, mice have lower rates of circulating neutrophils (10–25%) than humans (50–70%) and do not express defensins [11]. The baseline values and the names of neutrophil chemokines differ between rodents and humans, e.g., keratinocyte-derived chemokine in mice versus interleukin-8 in humans. Inter-species differences also exist between humans and pigs and/or piglets. Although the hemodynamics in humans and pigs are similar, the pulmonary vascular response to hypoxia (hypoxic vasoconstriction) is more pronounced in pigs than in humans [12]. To date, no available animal model perfectly mimics all key aspects of human VILI or acute respiratory distress syndrome (ARDS) [8, 13]; nevertheless, current models in use can help us better understand the mechanisms of VILI and develop new therapeutic approaches to mitigate lung damage. Selecting the animal model that most adequately fits the corresponding research question is of utmost importance.

**Table 1:** Common models of ventilator-induced lung injury

Type of injury	Example reference	Species	Advantages	Disadvantages
High V_T_ (> 30 ml/kg), single-hit model	[18]	C57BL/6 (small animals)	Isogenic background Ability for genetic manipulation Ready availability of diverse tissues Endpoints achievable Extensive lung pathology Measures of lung edema and permeability	Short time experiments Minimal hemodynamics data Difficult to obtain samples for arterial blood gas analysis
LPS i.t. or i.p. + high V_T_ (> 30 ml/kg), double-hit model	[36]	Wistar rats (small animals)	Similar to above Exploration of pathogenesis (direct vs indirect causes) Investigation of predisposing conditions	Similar to above Minimal hemodynamics data Difficult to obtain samples for arterial blood gas analysis
Surfactant lavage + LPS i.v. and high V_T_ (> 30 ml/kg), double- or triple-hit model	[32]	Pigs or piglets (large animals)	Extensive lung and distal organ pathology Measures of lung edema and permeability Receive ICU supportive care Radiographic and/or positron emission tomography assessment of lung injury	Labor intensive Moderate reversibility of lung injury
Surfactant lavage, single-hit model	[57]	Pigs or piglets (large animals)	Inhomogeneous lung aeration, alveolar collapse, increased susceptibility to additional hits, responsive to mechanical ventilation interventions, alveolar recruitability Measures of lung edema and permeability Receive ICU supportive care Application of imaging techniques	Staff required Labor intensive Pronounced reversibility Light to moderate lung inflammation
Surfactant lavage + high V_T_ (> 30 ml/kg), double-hit model	[67]	Pigs or piglets (large animals)	Extensive lung and distal organ pathology Measures of lung edema and permeability Continuous invasive and noninvasive physiologic assessment Receive ICU supportive care Application of imaging techniques	Staff required Labor intensive Moderate reversibility of lung injury Moderate lung inflammation

*LPS* lipopolysaccharide, *i.t.* intratracheal, *i.p.* intraperitoneal, *i.v.* intravenous, *ICU* intensive care unit

There are additional factors that should be explored further in preclinical studies, such as sex, age, and VILI resolution. Recently, sex was not associated with VILI susceptibility in mice [14]. These findings support the inclusion of both sexes in experimental studies rather than restricting the use of animals of a single sex [15]. Considering that most patients who undergo invasive mechanical ventilation are ~ 60 years old [16], the association between aging organs and mechanical ventilation should be explored further in future preclinical studies. There is insufficient evidence about pulmonary repair mechanisms in experimental VILI. The process after lung injury may involve resolution of alveolar/interstitial edema and inflammation, structural cell proliferation, and extracellular matrix organization [17]. Moreover, modulation of the redox capacity by the Nrf2-ARE pathway has been shown to increase resilience against oxidative stress during injurious mechanical ventilation [18]. In addition, therapy using a conditioned medium obtained from bone marrow and cryopreserved umbilical cord mesenchymal stem cells was able to reduce stretch-induced inflammation and cell death, thus enhancing VILI resolution [19].

### Static ventilator variables associated with VILI

**Fig. 1:**
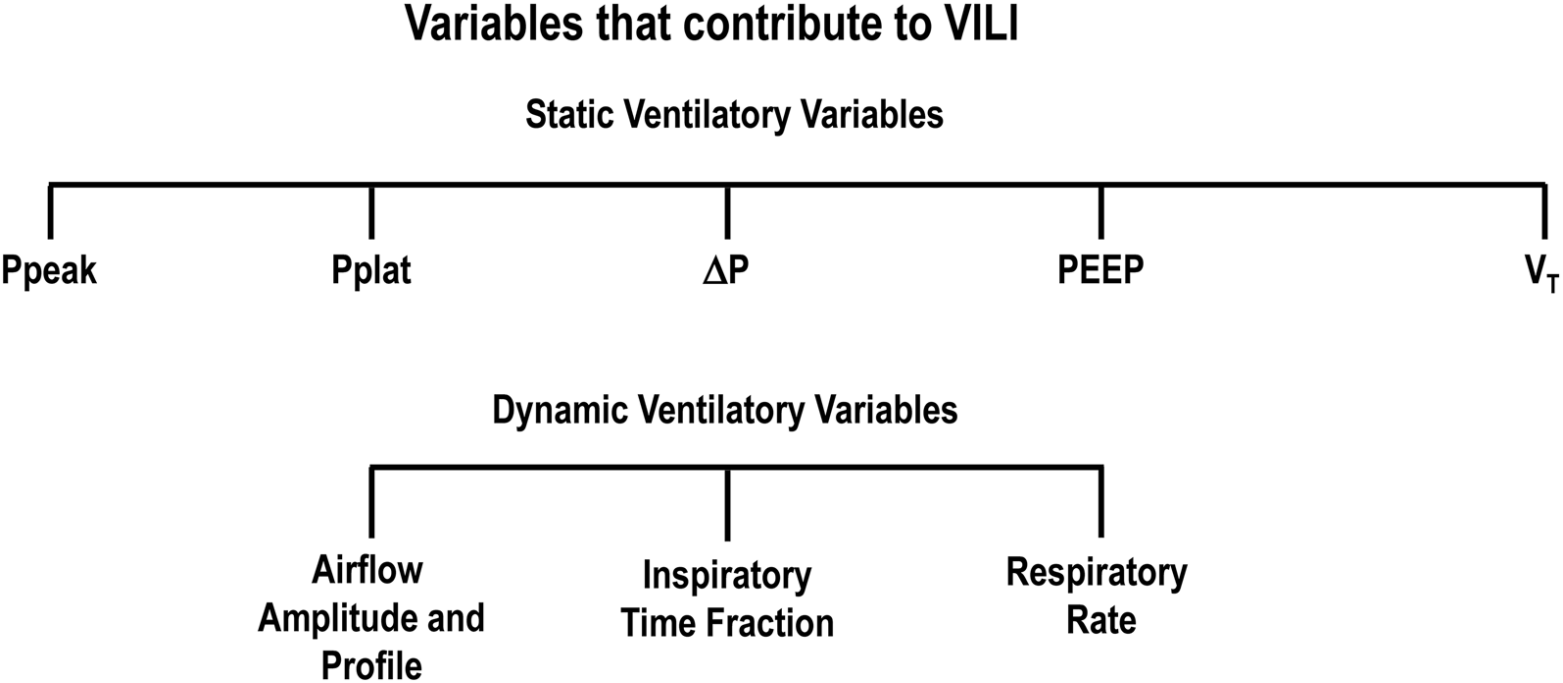
Static and dynamic ventilatory variables that contribute to ventilator-induced lung injury (VILI). *DP* driving pressure, *Ppeak* peak airway pressure, *PEEP* positive end-expiratory pressure, *Pplat* plateau airway pressure, *V*_*T*_ tidal volume

Peak airway pressure (Ppeak,_RS_), plateau airway pressure (Pplat,_RS_), positive end-expiratory pressure (PEEP), driving pressure (∆P,_RS_), and tidal volume (V_T_) are static ventilator variables associated with VILI (Fig. 1).

### Peak airway pressure

In pressure-controlled ventilation (PCV), Ppeak,_RS_ is the maximum pressure during inspiration and depends on the elastic and resistive components (airway, lung tissue) and equipment (endotracheal tube diameter and length) [20, 21]. PCV is usually associated with lower Ppeak,_RS_ compared with volume-controlled ventilation (VCV) due to the different flow profiles, but this difference is less important when the option of ramp flow is used in VCV. In 1974, Webb and Tierney [22] showed that healthy rats ventilated with high Ppeak,_RS_ (45 cmH_2_O) and zero PEEP presented perivascular and alveolar edema, lung overdistension, and barotrauma. On the other hand, a Ppeak,_RS_ of 45 cmH_2_O and PEEP of 10 cmH_2_O did not result in edema. In 2017, Katira et al. [23] reproduced the classic study of Webb and Tierney to clarify these different responses, focusing on heart–lung interaction in healthy rats. They showed that high Ppeak,_RS_ impairs right ventricular filling and pulmonary perfusion, resulting in right ventricular failure and dilation. This scenario is in line with endothelial cell injury and capillary stress failure, which may facilitate microvascular leakage of protein and water into the alveoli, yielding high permeability pulmonary edema. Thus, this preclinical study showed that increased Ppeak,_RS_ values should be avoided due to adverse heart–lung interactions.

### Plateau airway pressure

Pplat,_RS_ is calculated during a period when airflow is stopped at end inspiration and reflects end-inspiratory alveolar pressure. Pplat,_RS_ can be affected by changes in V_T_ and respiratory system compliance (C,_RS_) but not by changes in airflow and airway resistance [24]. The effects of four levels of Pplat,_RS_ (15, 20, 25, and 30 cmH_2_O) on alveolar-capillary barrier permeability to proteins were studied in a model of lung damage induced by hypertonic solution. Pplat,_RS_ between 20 and 25 cmH_2_O was associated with epithelial and endothelial cell damage as well as increased permeability [25].

Because Pplat,_RS_ can be affected by the properties of the chest wall, the chest wall component needs to be subtracted from the respiratory system, thus yielding the transpulmonary plateau pressure (Pplat,_L_) that is associated with the development of VILI. Limiting Pplat,_RS_ to ≤ 28 cmH_2_O was found to be effective in reducing the risk of overdistension and is widely accepted.

### Positive end-expiratory pressure

PEEP reflects the end-expiratory pressure remaining in the airways and, thus, the static preload of the respiratory system. The use of low PEEP levels may not be sufficient to reduce alveolar collapse and lung edema [26]. However, higher PEEP may cause lung overdistention in the more compliant areas of the lungs and hemodynamic impairment. How to best set the PEEP in experimental models of ARDS is still challenging and the following strategies have been described to date: PEEP titrated according to oxygenation, respiratory system compliance or driving pressure, transpulmonary pressure (esophageal pressure), and imaging (computed tomography scan, electrical impedance tomography) [27]. Nevertheless, there are controversies regarding the best PEEP to use in clinical ARDS; it should be set according to each patient considering lung function (arterial blood gases and mechanics), imaging findings (degree of recruitability), and phenotype (hypo- versus hyperinflammatory).

### Respiratory system driving pressure

∆P,_RS_ is defined as Pplat,_RS_-PEEP or V_T_ normalized to C,_RS_ [28], and ΔP,_L_ is defined as the difference between ∆P,_L_ at end inspiration and ∆P,_L_ at end expiration. ∆P,L can be calculated as:

$$\Delta {\text{P}},_{{\text{L}}} \, = \,\left( {{\text{Pplat}},_{{{\text{RS}}}} \, - \,{\text{P}}_{{{\text{ESO}},{\text{ end}} - {\text{insp}}}} } \right)\, - \,({\text{PEEP}}_{{{\text{TOT}}}} \, - \,{\text{P}}_{{{\text{ESO}},{\text{ end}} - {\text{exp}}}} ).$$

**Fig. 2:**
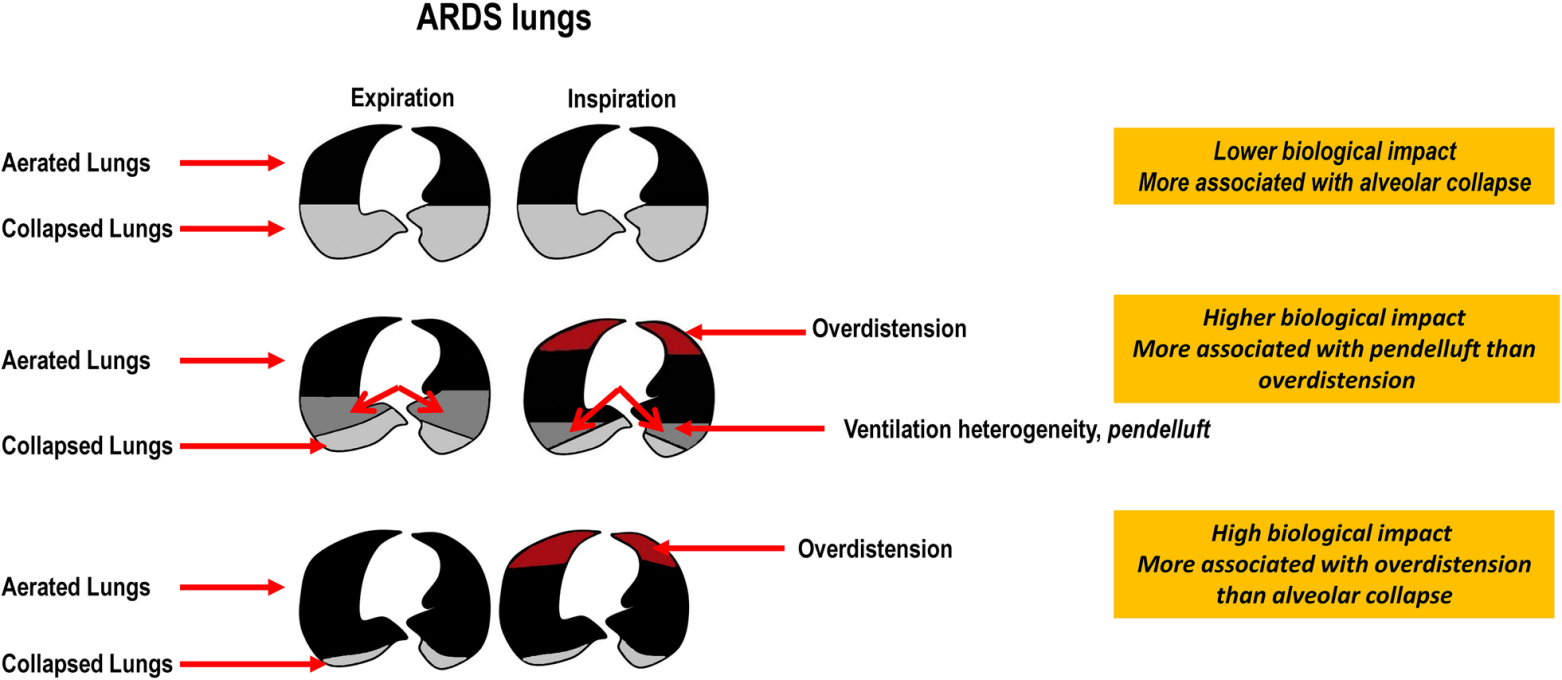
Lung morphology at expiration and inspiration in experimental ARDS, mechanically ventilated with low tidal volume (V_T_ = 6 ml/kg) and progressively increased positive end-expiratory pressure (PEEP). With low V_T_ and low PEEP, aerated lungs (baby lung) are ventilated and collapsed lungs are at rest. With progressive increase in PEEP, at low V_T_, areas of lung collapse reduce, areas of overdistension increase, and areas of alveolar lung heterogeneity and *pendelluft* arise; these areas are concentrated around the collapsed units, which present the highest lung stress. At the highest PEEP, the area of lung collapse reduces but even though lung overdistension remains increased, the degree of lung stress and the biological impact on lung tissue reduce because the area associated with *pendelluft* is no longer observed

Both ∆P,_RS_ and ΔP,_L_ have been shown to correlate positively with stress and strain [29, 30]. In experimental endotoxin-induced ARDS, different combinations of V_T_ and PEEP were used to create a range of ∆P,_L_. The combination of a V_T_ of 6 ml/kg and the lowest PEEP and ∆P,_L_ to maintain oxygenation within a normal range minimized VILI even in the presence of alveolar collapse [31]. In agreement with these results, Güldner et al. [32] observed that atelectrauma led to less inflammation than volutrauma strategies (Fig. 2). This strategy of keeping the collapsed lung closed is known as “permissive atelectasis”.

Static and dynamic ΔP,_L_ were compared in experimental ARDS. Using the same protective V_T_, pressure-support ventilation (PSV) resulted in similar static ΔP,_L_ but higher dynamic ΔP,_L_ compared with PCV, leading to higher expression of biomarkers associated with inflammation in PCV [33]. This preclinical study suggested that the main determinant of lung injury is, therefore, the static rather than dynamic ΔP,_L_.

### Tidal volume

Experimental models were also helpful in determining that overdistention rather than inspiratory pressure per se caused lung damage yielding volutrauma. In this context, Dreyfuss et al. [34] reported lung edema in animals ventilated with high V_T_ (40 ml/kg), but such edema did not develop when rats underwent ventilation with increased airway pressures with the use of straps around their abdomens and chests, which reduced the V_T_ (19 ml/kg).

Mechanical ventilation with low V_T_ (4–6 ml/kg) induces repetitive opening and closing of airways and lung units, promoting epithelial cell damage, hyaline membrane formation, and lung edema, which has been named atelectrauma [1]. Interestingly, considering the “baby lung” in ARDS, the shear stress in atelectatic areas induces less lung damage (4–5 times lower) than the force at the edges between aerated and atelectatic lung regions [31, 35].

Recently, Felix et al. [36] showed that in experimental ARDS, lung damage caused by high V_T_ (22 ml/kg) could be attenuated if V_T_ increased slowly enough to progressively (0.5 ml/kg/min) reduce mechanical heterogeneity and allow the epithelial and endothelial cells, as well as the extracellular matrix of the lung, to adapt. In contrast, extending the adaptation period (0.25 ml/kg/min) increased cumulative power and did not prevent lung damage.

### Dynamic ventilator variables associated with VILI

The dynamic ventilator variables associated with VILI are the RR, inspiratory airflow, and the inspiratory to expiratory time ratio (Fig. 1).

### Respiratory rate

Whereas V_T_ is set to match lung size, RR is usually set to maintain appropriate minute ventilation and meet the patient’s metabolic demand. In contrast to other ventilator variables, RR has been largely neglected compared with other potential variables that cause lung damage. However, when lungs are heterogeneously aerated, as shown in normal lungs [37] and a double-hit VILI model [38], high RR can amplify microstresses and regional strains, thus causing VILI. This phenomenon was shown to be modulated by the degree of pulmonary aeration [39]. The mechanisms of extracellular matrix, epithelial, and endothelial cell adaptation associated with different velocities of increases in RR were recently investigated in rats with experimental ARDS [40]. The animals received abrupt or different gradual increases of RR during protective ventilation. Longer RR adaptation resulted in less lung damage compared with abrupt RR increases. By promoting a gradual increase in RR, alveolar units remain open and better accommodate the stress (reduced airway pressures) for the same strain (V_T_). On the other hand, by promoting an abrupt increase in RR and shortening inspiratory time, only fast alveolar units remain open, which may favor alveolar overdistension, more heterogeneity, and lung damage. Thus, fast alveolar units, which better accommodate strain, tend to overdistend [41, 42]. After application of the recruitment maneuver, the fraction of slow alveolar units tends to decrease [43], as does the propensity of alveolar units to become atelectatic, which may decrease regional tidal strains and heterogeneity.

### Inspiratory airflow

The inspiratory airflow can also be adjusted in some modes of ventilation, which is also a potential cause of VILI [44]. The shear stress at the top of the cells within the respiratory bronchi increased injury. In this context, in situ experiments have shown that healthy lungs support magnitudes of shear stress (15 dyn/cm^2^) at all alveolar opening velocities in the physiologic range. However, for a lung with increased viscosity of intra-alveoli fluid, shear stress may increase by several orders of magnitude, enough to induce epithelial cell injury [45]. Some reports have associated high inspiratory airflow profiles with gas exchange, the work of breathing, cardiovascular function, and lung damage [46, 47]. Not only the inspiratory airflow amplitude can be harmful but also the airflow waveform (e.g., constant versus decelerating) may be a relatively neglected and modifiable determinant of VILI risk in ARDS [6, 48].

### Expiratory airflow: addressing expiration

Traditionally, less attention is given to the expiration phase than to inspiration during controlled mechanical ventilation. Nevertheless, the passive de-pressurization of the respiratory system in conventional ventilation modes predisposes closure of the distal airway and atelectasis formation. However, during so-called flow-controlled ventilation (FCV), airflows during inspiration and expiration are actively controlled and constant, whereas the airway pressure alternates between a peak and end-expiratory pressure, creating a triangular airway pressure profile [49, 50]. Thereby, FCV avoids zero-flow conditions. Along with physiologic improvements, FCV was shown to reduce VILI compared with conventional ventilation [49, 51]. Furthermore, Wittenstein et al. [50] showed that, regardless of fluid status, FCV reduced the mechanical power mainly due to the resistive component compared with VCV during one-lung ventilation. By actively controlling the expiratory phase, the appearance of intrinsic PEEP may be prevented, which in turn promotes better air exhalation among alveoli with different time constants. In a recent preclinical study, Busana et al. [52] studied healthy pigs randomized to a control group and a valve group, where the expiratory flow was controlled through a variable resistor, but all the pigs were ventilated under similar V_T_, PEEP, and inspiratory airflow. No differences were observed in respiratory mechanics, gas exchange, hemodynamics, wet-to-dry ratios, and histology, whereas the decrease in end-expiratory lung impedance was significantly greater in the control group compared with those that used the variable resistor. The authors concluded that the reduction in expiratory flow occurred mostly across the endotracheal tube and partly in the respiratory system. The beneficial effect of the variable resistor at the expiratory phase may also be dependent on heterogeneous and injured lungs at baseline [53].

### Effects of inspiratory to expiratory time ratios

In a model of mild ARDS, mechanical ventilation with increased inspiratory to expiratory ratios (2:1) led to increased gene expression of biological markers associated with inflammation and alveolar epithelial cell damage, whereas a reduced inspiratory to expiratory ratio (1:2) increased markers of endothelial cell damage, and an inspiratory to expiratory of 1:1 minimized lung damage [54]. Similar results were observed in another preclinical study using high V_T_ and prolonged inspiratory time [55].

### Mechanical power as a hub for the development of VILI

Mechanical power (MP) is the mechanical energy delivered from the ventilator to the respiratory system and has been considered to be a unifying driver of VILI [49–51].

The following formula for MP was described in 2016 [56]:

$${\text{MP}}\, = \,\left( {\Delta {\text{V}}^{{2}} \, \times \,\left[ {\left( {0.{5}\, \times \,{\text{E}},_{{{\text{RS}}}} \, + \,{\text{RR}}\, \times \,\left( {{1}\, + \,{\text{I}}:{\text{E}}} \right)/{6}0\, \times \,{\text{I}}:{\text{E}}\, \times \,{\text{Raw}}} \right)\, + \,\Delta {\text{V}}\, \times \,{\text{PEEP}}} \right]} \right)\, \times \,{\text{RR}}.$$

Not all combinations of the three pressure components of energy (elastic, resistive, and PEEP components) and RR are equally hazardous. Doubling RR increases MP by 1.4-fold, doubling PEEP increases MP by twofold, whereas doubling V_T_ increases MP by fourfold [49]. The increase in transpulmonary MP has been associated with the development of VILI [50]. Moreover, even at low V_T_, high MP promoted VILI [51]. In short, all combined variables of MP must be considered together [51, 52]. In a study of experimental ARDS in pigs, MP was positively correlated with pulmonary neutrophilic inflammation, which is a mainstay of ARDS pathogenesis [57]. Different formulas for MP have been described [58]. The most simplified version is based on the classic equation of motion:

$${\text{MP}}\, = \,0.0{98}\, \times \,{\text{V}}_{{\text{T}}} \, \times \,{\text{RR}}\, \times \,({\text{Ppeak}},_{{{\text{RS}}}} \, - \,\Delta {\text{P}},_{{{\text{RS}}}} /{2}).$$

This formula computes three components, i.e., static PEEP × volume, elastic, and resistive; other formulas compute only the elastic and/or resistive component [59]. Whether the static PEEP × volume component should be included in the MP formula or not is a topic of intense debate [59, 60].

Another point of debate is whether a single measurement of MP in a short time frame (e.g., 1 min) would be clinically meaningful compared with computing the cumulative MP over a relevant time frame, likely better reflecting the time in which subjects are exposed to a certain MP. The cumulative MP has been proposed in preclinical studies [36]. In practical terms, it would be feasible to include the cumulative MP variable at the trends window available in different types of mechanical ventilators. The cumulative MP would reflect (1) all the MP values since the first minute of invasive mechanical ventilation; (2) not only the total amount of MP delivered to patients’ lungs but also how fast the MP is applied; (3) recognition if the injuring strain threshold has been breached [48]; (4) the big panorama of the most injurious ventilator variables, which up to now are not well recognized, such as minute ventilation; (5) the ratio between measured and expected MP [61]. Whether MP, cumulative MP, or MP normalized to lung volume (i.e., intensity) better reflects or predicts VILI is as yet unclear but is currently under investigation in different experimental studies [58, 59].

### Asynchronous subject-ventilator interaction as a factor in VILI

Previously, severe subject-ventilator asynchrony (SVA), i.e., mismatch between the patient respiratory effort and the ventilator support provided, was shown to be associated with worsened clinical outcomes of patients in the intensive care unit and suggested to be causally linked to VILI by increasing transpulmonary pressure and *pendelluft* [62–64]. However, it remained unclear if severe SVA directly contributed to VILI or if it was a symptom or marker rather than a causal factor [65, 66]- This was investigated in a study on mechanically ventilated pigs with experimental ARDS. In that study, SVA (ineffective, auto-, or double-triggering) was actively provoked by random variation of respiratory variables and compared with both assisted and controlled ventilation. It was found that 12 h of severe SVA did not increase lung injury as assessed by histology or by biomarker expression [67], questioning the concept that SVA is directly linked to VILI, at least if lung-protective ventilator settings are respected. However, a different recent study showed contrasting results. In surfactant-depleted rabbits, SVA was induced by phrenic nerve stimulation [68]. Breath stacking was associated with high V_T_ and inspiratory lung stress and yielded both lung and diaphragm injury, and reverse triggering caused diaphragm injury. The discrepancy from the previous study may be explained by different methodological approaches and ventilator settings, especially regarding PEEP. The role of SVA regarding VILI and clinical outcomes warrants further investigation, but the current literature suggests that SVA may not necessarily directly induce VILI.

### Future role of experimental studies in intensive care medicine

In the last decades, experimental research has fostered the development of modern intensive care. Experimental studies allow the use of new methods and measurements to effectively investigate lung physiology and pathophysiology. Important mechanisms, such as “lung rest” and the “baby lung concept” [69], have been elucidated based on experimental research, which was then translated to the clinical setting. In contrast, some interventions and concepts that significantly improved physiologic variables in animal studies did not translate into substantially improved clinical outcomes [70]. Currently, there is a tendency toward outcome- and epidemiology-oriented clinical studies with large sample sizes [69]. In addition, social and political movements challenge the need for experimental research. A European Citizen’s Initiative (ECI) recently called for the complete abolition of all animal testing in research in the EU [71], posing an acute and direct risk for animal research in intensive care. After a public hearing, plenary debate in the European Parliament, and cautious analysis, the European Commission recently responded by emphasizing that animal models are currently unavoidable to investigate complex biological or physiologic processes [71].

Accordingly, animal models will continue to play an important role for scientific progress in intensive care, especially because they allow interventions that would not be possible or ethical in humans in potentially life-saving medical interventions. For example, animal models enabled the investigation of actively induced SVA [67] as well as the extensive use of lung imaging techniques in controlled pathophysiologic conditions [72]. Animal studies will continue to allow the necessary translational approach, in which research questions and concepts may be developed in the preclinical setting and transferred to clinical studies to improve intensive care approaches. Research questions and hypotheses will then be derived from the clinical routine and continuously investigated in the experimental setting. For respiratory and mechanical ventilation research, which usually requires an intact cardio-respiratory system, animal experiments may continue to be needed, at least as long as valid alternatives, such as organoids, are not available.

So far, valuable lessons have been learned on the basis of experimental VILI models. However, further open research questions require careful investigation under controlled conditions, which can be optimally achieved with the help of animal models (Table 2). This again emphasizes the future role of experimental research in intensive care medicine.

**Table 2:** Lessons learned from experimental studies and future experimental objectives

What is known?	Mechanisms to be explored with preclinical research
Increased Ppeak,_RS_ can impair heart–lung interaction	Microvascular injury mechanism followed by increased permeability to water, proteins, or even cells Improve methods or techniques to non-invasively monitor pulmonary vascular dynamics (echocardiography, electrical impedance tomography, among others) Easily identify conditions for the risk of lung injury
Pplat,_RS_, between 20 and 25 cmH_2_O, is associated with epithelial and endothelial cell damage as well as increased permeability	Further development of participation of interstitial compartment modeling, according to Pplat,_RS_ levels Development of permeability markers to be used in preclinical studies with potential to be used in clinical studies, according to Pplat,_RS_ levels Further explore the participation of the chest wall in different scenarios, such as obesity, pneumoperitoneum, and abdominal compartment syndromes
Low PEEP levels may not be sufficient to prevent alveolar collapse and lung edema, whereas high PEEP may cause lung overdistention and hemodynamic impairment	Further evaluate the effect of PEEP in a “personalized approach” taking into account individual lung mechanics, imaging, and/or ARDS phenotypes
The static, but not dynamic, ΔP,_L_ represents the main determinant of lung injury	Evaluate the diaphragm and cardiovascular consequences of increased and decreased levels of ΔP,_L_ What are the biotrauma consequences of increasing levels of ΔP,_L_ Further evaluate ΔP,_L_ during spontaneous breathing, because it can be very high and it is considered one of the mechanisms of P-SILI
Epithelial, endothelial cells, and the extracellular matrix of the lung may adapt to increasing levels of V_T_	At the micro-scale, further evaluate the adaptation of structural cells to static and cyclic stretch, associated or not with endotoxin Study the increasing V_T_ taking into account the mechanical power and cumulative mechanical power generated, by changing the time under MV Evaluate alveolar-capillary barrier resolution and how lipids and water accommodate increasing V_T_
Gradually increasing respiratory rate may be beneficial for abrupt increases with regard to lung aeration and accommodation of stress for the same V_T_	Further investigate if the lung damage after abrupt increases in RR is dependent on alveolar heterogeneity at baseline Investigate the importance of mechanical power measured within 1 min and the cumulative mechanical power within a long time scale Confirm whether a threshold of transition to injury exists to precisely determine the pace of gradually increasing RR Refinement by imaging studies that show in real time what happens to the number of fast alveolar units
High inspiratory flows and flow profiles may be associated with lung damage	At micro-scale, evaluate the mechanosensing and transduction of shear stress on structural cells and how they may adapt depending on the magnitude and profiles Evaluate heart–lung interaction under increased magnitude of airflow because it can also increase Ppeak,_RS_
Mechanical power is associated with VILI	Mechanical power, cumulative mechanical power, intensity – which is the best predictor of VILI?

*PEEP* positive end-expiratory pressure, *ARDS* acute respiratory distress syndrome, *MV* mechanical ventilation, *RR* respiratory rate, *VILI* ventilator-induced lung injury

## Conclusion

Preclinical studies using animal models have advanced understanding of the mechanisms of VILI, thus stimulating strategies to mitigate lung damage in patients with ARDS. Even in times of large epidemiological clinical trials, computer modeling studies, and the trend toward abolition of animal testing, innovation, and progress in respiratory and ventilation research, are still based on necessary experimental studies in small and large animals. These studies allow the interpretation of VILI mechanisms and have shown that static and dynamic components are essential variables, which must be controlled by the operator. According to the different models of VILI associated with ARDS: keep the lungs partially collapsed (SaO_2_ > 88%), avoid opening and closing collapsed alveoli, and gently ventilate aerated regions while keeping collapsed and consolidated areas at rest. Additional mechanisms, such as SVA, cumulative power, intensity, as well as damaging strain threshold (stress–strain level at which tidal damage is initiated), are under experimental investigation and may enhance the understanding of VILI.

## Data Availability

Not applicable.

## Abbreviations

ΔP,_L_: Transpulmonary driving pressure
ΔP,_RS_: Driving airway pressure
ARDS: Acute respiratory distress syndrome
FCV: Flow-controlled ventilation
MP: Mechanical power
PCV: Pressure-controlled ventilation
PEEP: Positive end-expiratory pressure
Ppeak,_RS_: Peak airway pressure
Pplat,_L_: Plateau transpulmonary pressure
Pplat,_RS_: Plateau airway pressure
PSV: Pressure-support ventilation
RR: Respiratory rate
SVA: Subject-ventilator asynchrony
VILI: Ventilator-induced lung injury
V_T_: Tidal volume
